# IART: Inertial Assistant Referee and Trainer for Race Walking †

**DOI:** 10.3390/s20030783

**Published:** 2020-01-31

**Authors:** Teodorico Caporaso, Stanislao Grazioso

**Affiliations:** Fraunhofer Joint Lab IDEAS, Department of Industrial Engineering, University of Naples Federico II, 80125 Naples, Italy; stanislao.grazioso@unina.it

**Keywords:** race walking, inertial sensor, user-centered design, biomechanics, training improvement, judgment, step classification

## Abstract

This paper presents IART, a novel inertial wearable system for automatic detection of infringements and analysis of sports performance in race walking. IART algorithms are developed from raw inertial measurements collected by a single sensor located at the bottom of the vertebral column (L5–S1). Two novel parameters are developed to estimate infringements: loss of ground contact time and loss of ground contact step classification; three classic parameters are indeed used to estimate performance: step length ratio, step cadence, and smoothness. From these parameters, five biomechanical indices customized for elite athletes are derived. The experimental protocol consists of four repetitions of a straight path of 300 m on a long-paved road, performed by nine elite athletes. Over a total of 1620 steps (54 sequences of 30 steps each), the average accuracy of correct detection of loss of ground contact events is equal to 99%, whereas the correct classification of the infringement is equal to 87% for each step sequence, with a 92% of acceptable classifications. A great emphasis is dedicated on the user-centered development of IART: an intuitive radar chart representation is indeed developed to provide practical usability and interpretation of IART indices from the athletes, coaches, and referees perspectives. The results of IART, in terms of accuracy of its indices and usability from end-users, are encouraging for its usage as tool to support athletes and coaches in training and referees in real competitions.

## 1. Introduction

Wearable technologies in sports applications are useful tools for measuring the athlete’s performance in outdoor conditions, and thus they can play a relevant role to support training. Moreover, as they can provide accurate and reliable data from the athletes, they can also be used for developing tools able to support judgments.

**Figure 1:**
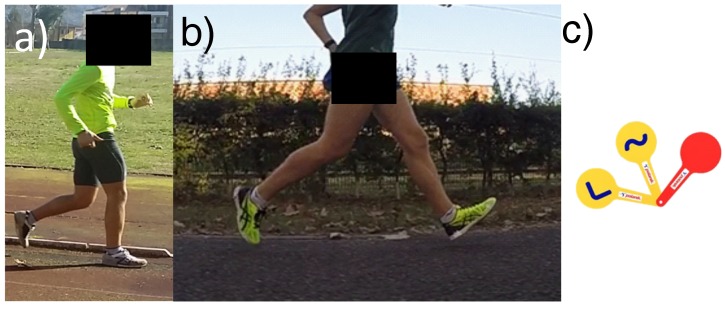
Infringements in race walking: (**a**) athlete in bent knee infringement (with a flexion of the knee before the vertical upright position) and (**b**) athlete in LOGC infringement; (**c**) yellow paddles with the two symbols associated with the two infringements (“<”: bent knee; “∼” LOGC) and red paddle (associated with the disqualification of the athlete).

A sport discipline in which wearable tools can be used for both performance and infringement assessment is race walking, a historical long-distance discipline within the athletics program. The rule 230 of World Athletics Competition rule [1] defines the race walking as “a progression of steps so taken that the walker makes contact with the ground, so that no visible (to the human eye) loss of contact occurs. The advancing leg must be straightened (i.e., not bent at the knee) from the moment of first contact with the ground until the vertical upright position.” According to this rule, two infringements are thus possible: bent knee and loss Of ground contact (LOGC), see Figure 1 for a graphical interpretation.

In the race walking context, the main users are (i) athletes, leading actors of the race; (ii) coaches, “chief technical officers” of the athletes’ team; and (iii) judges, guarantors of the regularity of the race. All these stakeholders are currently interested in novel tools for monitoring the sports technique in training and competition scenarios. Athletes are interested in having objective feedback about their performance and technique. Coaches are interested in having key indicators of the performance and infringements of athletes, useful for developing new customized strategies to optimize training and competitions. Judges are indeed interested in useful tools to assist their evaluation of infringements during races. With this respect, as the LOGC is the most common infringement in competition scenarios [2], having systems able to estimate and detect this value in a reliable manner during competitions would be of great interest. It is also important to note that the LOGC lasts a few hundredths of a second, so it is highly challenging to evaluate it (reliably) using only human eyes, as mostly happens in the current practices [3]. The importance of this need is also underlined by the international federation (i.e., World Athletics), which is interested in the definition of a novel competition system able to evaluate the LOGC, to reduce the issues connected with judgment and therefore improving the outside credibility of race walking.

In field conditions, four technologies are potentially available for estimation of LOGC: high-speed camera, optical measurement systems, insole pressure, and wearable inertial systems. Video analysis using high-speed camera provides reliable evaluation of sports kinematic parameters; indeed, several authors [4,5] have used this technology for the assessment of LOGC. However, video analysis requires an intensive postprocessing and therefore it is difficult to use in real conditions (training and competition scenarios), where a continuous and real-time evaluation is required. In summary, the basic limitations of video analysis are (i) it is a time-consuming process and (ii) it does not allow a continuous analysis of the athletes, especially where the athletes are in group. Recently, optical measurement systems (i.e., OptoJump Next system) have been used for race walking analysis [6,7]. In particular, the authors of [6] demonstrated how this system can be used to provide highly reliable values for assessment of LOGC timing in elite race walkers, and in overground and treadmill testing. This technology allows a faster evaluation of LOGC, if compared to video analysis. However, even this technology is difficult to use in real training and competition scenarios as it requires the athlete walking alone, and it allows to analyze only few steps. The use of an insole pressure system is in progress by Amigo ([8], under World Athletics investigation). The system is composed by piezoelectric insole pressure sensors (with thickness lower than 1 mm), which collect LOGC data that are then transmitted to a control unit by R-FID (radio-frequency identification). The insole system allows a direct measure of the LOGC, but it could be invasive from the athlete’s perspective. Indeed, the direct contact with the foot could lead to problems (e.g., foot blisters), in particular for the long-distance competitions typical of race walking. The use of wearable inertial systems, even if they do not offer a direct assessment of gait temporal events, can potentially reduce the discomfort of the insole pressure systems, resulting more user-friendly in real training and competition scenarios. The first usage of inertial sensors in race walking dates back to the work proposed by Lee et al. in [9]. They used a single inertial sensor (with sample frequency of 100 Hz) placed on S1 vertebra, to correlate acceleration patterns with gait temporal events, for assessment of LOGC. The experimental validation was carried out by involving seven Australian race walkers. Over 80 collected steps, their accuracy in the inertial-based detection of LOGC events was equal to 91%, using values from video analysis as benchmark. More recently, authors in [10] presented a method based on machine learning algorithms for identification of race walking infringements (LOGC and bent knee). They started to test a system composed by seven inertial sensors (with sample frequency of 60 Hz) and they involved eight experts Italian race walkers. A total of 972 strides (i.e., 1944 steps) were collected. Starting from data collected by four different body segments, three different combinations of signals for each body segment were elaborated by using nine different machine learning algorithms (for a total of 108 classifiers). The validation of classifiers was carried out using the judgment evaluation of a coach as benchmark. The study shows how the classifier based on the quadratic support vector machine fed with the shank linear acceleration gives the best performance with an overall accuracy value equal to 93%, with respect to a subjective evaluation of a coach.

Despite the good results achieved so far in the previous works, their major limitations can be summarized as follows, none of them consider specific requirements and characteristics of real competition scenarios, as recommended by World Athletics; they do not consider the involvement of elite athletes, and thus they do not derive elite-oriented assessment of infringements; they only provide estimation of infringements, without considering the performance parameters. As consequence of the last limitation, none of the previous work can be used to support both judges and coaches during competitions and training, respectively.

This work presents IART, the Inertial Assistant Referee and Trainer for race walking. The IART system offers tools for estimation of infringements and evaluation of performance in race walking, through indices customized for elite athletes. The preliminary development and results of the system have been presented in the conference paper [11]. With respect to this previous work, here we present a different method for estimation/classification of LOGC and an extensive study to validate the proposed indices for evaluation of infringements and performance. The overall contribution of the current work are (i) novel method for estimation of LOGC timing based on elite athlete’s kinematics, (ii) novel classification method for LOGC events based on eye limits and World Athletics competition rules, (iii) intuitive radar chart representation of infringements and performance indices, and (iv) validation of the results with a large number of elite athletes. The rest of the paper is organized as follows. In Section 2, we describe in details the methods used for derivation and validation of the main contributions of the work, as well as the experimental protocol. Then, in Section 3, we show the results and provide an extensive discussion. Finally, Section 4 reports the conclusions. Three appendices are used to provide additional details of the methodology and results.

## 2. Materials and Methods

### 2.1. Experimental Protocol

The experimental protocol involves the description of the participants, the experimental set-up and the procedure for data collection.

#### 2.1.1. Participants

Nine elite race walkers (seven men and two women) from three different countries participated to the experimental validation. They were specialists of 20 km (three men and the two women) and 50 km (four men). All race walkers were members of their national team; seven race walkers possessed the world championship entry standard for London 2017 (84 min for 20 km men, 96 min for 20 km women, and 244 min for 50 km men) and the other two possessed a personal best close to entry standard. The race walkers were informed about all the tests and possible risks involved, and they provided an informed consent before testing according to the Statement of Ethics Committee of University of Naples Federico II (Ref. Protocol 403/19). The participants did not have injuries and illness in the testing day and did not have experience of severe injuries in the previous twelve months. The test leader collected race walkers’ personal details (i.e., personal best on 20 km with respect to the best results achieved by the athlete in the last two seasons, age, and experience) and anthropocentric characteristics (i.e., stature); the mean and standard deviation values of these parameters are reported in Table 1.

#### 2.1.2. Experimental Set-Up

**Figure 2:**
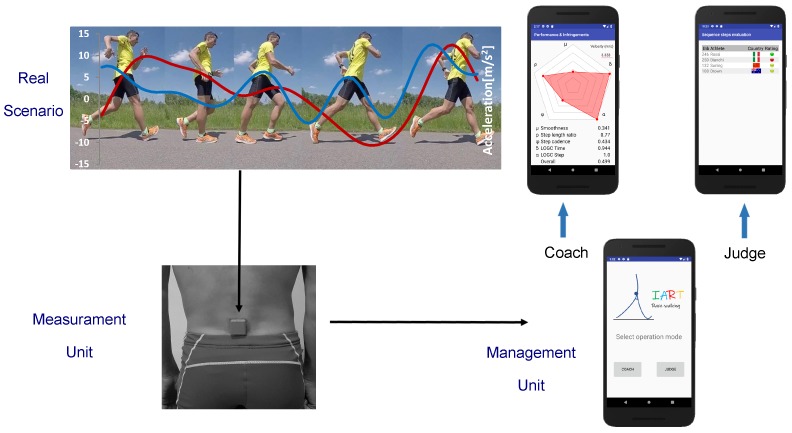
Basic architecture of IART. On the top-left, photo sequence of a real scenario with the acceleration data of CoM (red, vertical; blue, anteroposterior). On the bottom-left, the measurement unit placed at the end of athlete’s column vertebra. On the right, the management unit with a focus on the two modes of functionalities, as shown on the mobile app: coach mode and judge mode.

The experimental set-up is based on the results of our previous work [12], where the Kansei engineering method was used to select the optimal architecture of IART from the end-user’s perspective. Indeed, this method allows to consider in the design process the internal sensations from the end-users, and translate them into technical requirements or design elements [13]. In this phase, we involved 50 end-users divided into three selected group (50% athletes, 30% trainers, and 20% judges) with an international experience. The objective was to select the placement of the inertial sensor on the human body and the functional specifications of the IART system. From this study, we developed the conceptual architecture reported in Figure 2. The measurement unit is placed at the bottom of the race walker’s vertebral column, whereas the management unit is a mobile app designed for the main users (athletes/coaches and judges). According to this concept, we select a measurement unit able to provide the following functional requirements: (i) sample frequency and dynamic range adapted to achieve a good reliability in relationship with the characteristics of the race walking gesture for the assessment of infringement and performance parameters), (ii) minimal invasiveness for the athletes, and (iii) autonomy at least equal to 4h/5h hours (the maximum duration of the longer competition distance, 50 km). With this in mind, we chose the model type G-Sensor2 (BTS Bioengineering, Milan, Italy), an inertial sensor with the technical features reported in Table 2. The inertial sensor transmits over Bluetooth the collected data to a management unit installed on a mobile device.

#### 2.1.3. Data Collection

The inertial sensor was placed at the end of the athlete’s spine, on the spinous processes of L5–S1 vertebrae. The sensor placement process started by palpating the posterior superior iliac spines, then the athlete was asked to perform an anterior bending in order to better individuate the two upper spinous processes. The collected inertial data were transmitted to a mobile device mounted on a bicycle that followed the athlete during the trials. The bicycle was also equipped with the high-speed camera GoPro Black Hero 4 (Woodman Labs, San Mateo, CA, USA). The configuration used for the camera was “Super Slow Motion: WVGA” (240 fps) to provide a reliable and accurate benchmark for the inertial system. This configuration offers a low-resolution video (848 × 480 in 16:9); however, it is the best configuration for super slow motions (required for LOGC camera assessment). The high-speed camera was fixed on the rear dropout of the bicycle and controlled remotely via wireless connection by a mobile device positioned on the handlebars.

Trials were performed on a long-paved road, straight and flat in accordance with the World Athletics recommendations about race walking courses [1]. The tests were carried out in well-lit conditions, to have high-quality videos. After a standard self–selected warm up of 15 min (including also mobility exercise), the athletes performed 4 trials of 300 m each, at different incremental mean speeds (from 12.0 km/h to 14.5 km/h). These speeds allowed to cover, for each race walker, a range from 93% to 100% of the athlete’s personal best racing pace on 20 km. For the speeds between 12.0 km/h and 14.0 km/h, the speed incremental gain was fixed equal to 1.0 km/h; then, it became 0.5 km/h. Tests with a difference over ±0.2 km/h (for the speed from 12.0 km/h to 14.0 km/h) and over ±0.1 km/h (for the speed from 14.5 km/h) were excluded from the evaluation. In addition, to collect data also in the range of higher speeds, the two specialists on 20 km performed two additional tests at the following speeds: 15.0 km/h (±0.1 km/h) and 15.5 km/h (±0.1 km/h). The test run order of each athlete was randomized. To monitor the correct pacing, every 50 m on the road were signed and the test leader, by using a GPS watch, controlled the performance (checking the mean speed). A rest time of 90 s was set between two consecutive trials, to allow the race walker to recover.

### 2.2. Data Processing and Analysis

Here, we include the processing and analysis of raw data collected from the inertial sensor to derive the infringements and performance parameters for elite race walkers. First, we present the development of the infringements parameters, which we consider as the major contributions of the current work. The LOGC timing ($LOGC_{T}$) parameter is derived from acceleration data, by proposing a modification to a previous approach [9]. Starting from the $LOGC_{T}$, three different LOGC classification methods have been derived, respecting the race walking competition rules. Afterwards, we present the performance parameters used for analysis of race walkers: step cadence, step length ratio, and smoothness. Differently from the infringements parameters, the performance parameters are taken from literature. A graphical summary of the proposed methodology is given in the flow chart of Appendix A.

#### 2.2.1. LOGC Timing

The $LOGC_{T}$ computation is carried out, starting from the acceleration data collected by the inertial sensor. The first phase is filtering of accelerations. We have used a fourth-order Butterworth low-pass filter with a cut-off frequency of 20 Hz for acceleration on *x*-axis (i.e., the vertical acceleration of CoM) and 30 Hz for acceleration on *z*-axis (i.e., an approximated value of the anteroposterior acceleration of CoM). This filter with a cut-off frequency of 20 Hz is the same used by previous related works (race walking tests using inertial sensors) [9,10]. For the *z*-axis, we have indeed selected a cut-off frequency of 30 Hz, to consider more details of the original signal. We have verified that over 70% of the signal is lower than the cut-off frequency. In order to delete the phase shift, the signals were filtered two times.

**Figure 3:**
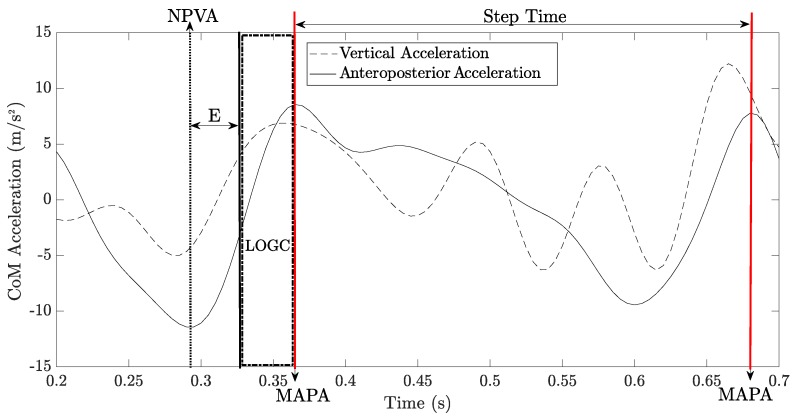
The correlation between the approximated CoM accelerations and temporal events (NPVA: temporal instant of negative peak vertical acceleration; MAPA: temporal instant of maximum anteroposterior acceleration). The temporal distance *E* is the threshold value for loss of ground contact (LOGC) assessment. The temporal distance between two following MAPA represents the step time. Notice that, differently from Equation 1, we have used the following nomenclature; NPVA = $t_{{min}_{i}}$; MAPA = $t_{{max}_{i+1}}$.

The $LOGC_{T}$ is defined as the time that elapses between the last instant of foot contact during the stance phase, called toe-off event (TOE), and the instant in which the following foot first makes contact with the ground, called heel-strike event (HSE). For the assessment of $LOGC_{T}$, we start from the definition in [9], where the $LOGC_{T}$ is defined as the time interval after which we can consider that the “flight is deemed to have occurred”. Therefore, it is not strictly defined as “the duration of loss ground contact”. According to [9,14], for the *i*-th step, the $LOGC_{T}$ expression is equal to

(1) $$LOGC_{T_{i}}=t_{{max}_{i+1}}-t_{{min}_{i}}-E_{i}$$

where $t_{min_{i}}$ is the temporal instant of minimum vertical acceleration of the current step, $t_{max_{i+1}}$ is the temporal instant of HSE at the successive step (as seen on the anteroposterior acceleration profile), and $E_{i}$ is a threshold value. Figure 3 shows a graphical interpretation of the LOGC timing and step time, by plotting the approximated CoM accelerations as function of time. Notice that the LOGC value in Equation (1) refers to a single step. However, according to the World Athletics regulations, for evaluation of infringement, the judges must consider a sequence of steps instead of a single step. The calculation of the number of steps (NS) to be included in a step sequence for analysis of infringements is reported in Appendix B: starting from the estimation of judge’s field of view in real competition scenarios, we obtain NS equal to 30 steps. The mean of the 30 values of loss of ground contact timing for a step sequence is computed as

(2) $$LOGC_{T,S}=\frac{1}{NS}\sum_{i=1}^{NS}LOGC_{T_{i}}$$

In the following, we refer to $LOGC_{T}$ in Equation (1) when we consider a single step and we refer to $LOGC_{T,S}$ in Equation (2) when we consider a step sequence. For simplicity, when we consider a single step, we omit the subscript *i* for all parameters.

**Figure 4:**
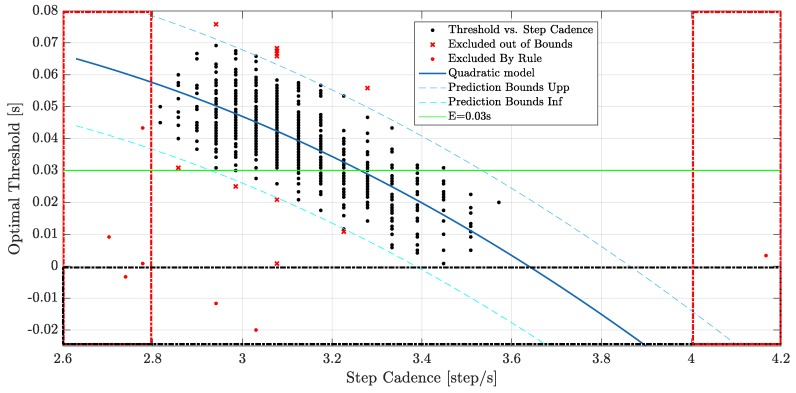
Scatter plot of optimal threshold values as function of the step cadence. Areas highlighted by red and black dash-dot lines are excluded from the analysis, respectively, for step cadence and for optimal threshold values.

**Figure 5:**
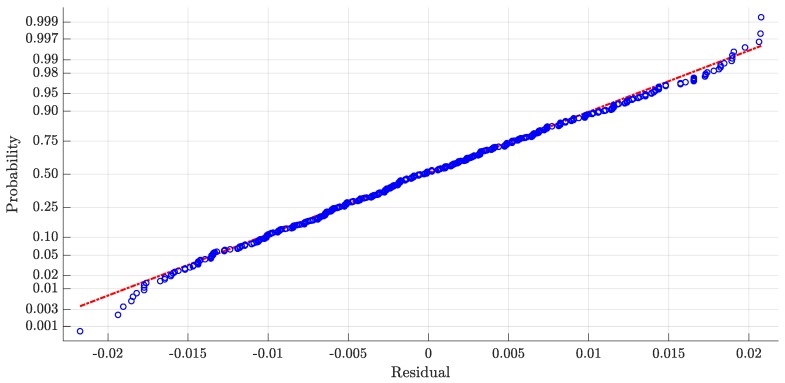
Plot of normal probability of residuals (response optimal threshold).

The threshold value *E* in Equation (1) was fixed to three hundredths of a second (0.03 s) in [9]. However, as shown in our previous work [2], with this fixed value for *E*, the mean difference between $LOGC_{T}$ and $LOGC_{B}$ (i.e., the benchmark value obtained from video analysis) decreases when the speed increases (from 20 ms at the speed of 12.0 km/h to 5 ms at speed of 14.6 km/h). This trend could be connected to a wrong value of this threshold; therefore, we propose to define the threshold value *E* as function of the speed. For the definition of the novel threshold *E*, we start from previous biomechanical research and our experimental data. In [15], pooling together data from 11 different studies, the authors show a linear descriptive equation between the step cadence (SC) and the race walking speed. This means that speed and SC are correlated. Therefore, we reanalyze the data related of 720 steps of experimental phase of the previous work, and we carried out a regression model between the optimal threshold (OT) time for each step (*E* value such that the time difference between $LOGC_{T}$ and $LOGC_{B}$ is equal to 0) and the corresponding SC. Starting from the experimental data, we excluded data that were clearly wrong, as they were (i) outside the normal range of step cadence (SC < 2.8 step/s and SC > 4.0 step/s), (ii) with OT < 0 (because the TOE is surely after the bottom of vertical acceleration), and (iii) outside the bounds of 99% of the model. In this way, we excluded from the regression analysis: 8 steps regarding the points (i) and (ii); 11 steps regarding point (iii). We choose the quadratic model without constant correlation that performed the best statistical index (R-Squared over 98% with the least-angle regression (LAR) and R-Squared of 60.1% with respect to standard regression analysis). For the *i*-th step, the novel threshold value $E_{i}$ is defined with the following quadratic model,

(3) $$E_{i}=\frac{1}{aSC_{i}^{2}+bSC_{i}}$$

where $SC_{i}$ is the step cadence for the *i*-th step (expressed in steps/s) which is defined in Section 2.2.3. The parameters *a* and *b* were fixed and, respectively, equal to a=−40.921 and b=11.242. Figure 4 shows the scatter plot between OT and SC. From the plot, we can see that the fixed threshold *E* equal to 0.03 s does not correlate with the real data collected from the athletes. Indeed, the fixed value of *E* only fits few real data. Indeed, the quality of the regression analysis, and the quadratic expression of *E* (on LAR), is demonstrated by a R-Squared value greater than 98% (see Table 3); the good agreement between the derived model and experimental data is also demonstrated by the normality of residual plot (see Figure 5).

#### 2.2.2. LOGC Classification

During race walking competitions, the judges are located along the circuit in a position that allows monitoring the race walkers in a specific part of the circuit. They can judge the athlete’s technique as (i) “legal” (i.e., no action is required); (ii) “doubt”, giving the race walker a yellow paddle (i.e., a warning); and (iii) “illegal”, giving the race walker a red card (i.e., proposal for disqualification).

In the following, starting from the computation of both values of LOGC timing ($LOGC_{T}$ and $LOGC_{T,S}$) and the assessment of the judge’s field of view (see Appendix B), we propose three different strategies for classification of steps and step sequences: binary, three-levels, and fuzzy classifications. In particular, the step sequence classification of LOGC is the most interesting from the regulation point of view, as the World Athletics regulations state that the judges must consider a sequence of steps instead of a single step for evaluation of infringements. The three-levels and fuzzy classifications are introduced to consider the “doubt case” of infringements, corresponding to the yellow paddle of the judge. For each step sequence, we define the loss of ground contact classification parameter ($LOGC_{C,S}$) as

(4) $$LOGC_{C,S}=\frac{\mathrm{Illegal}\;\mathrm{steps}}{NS}$$

##### Binary Classification

The procedure assigns each step (or each step sequence) to the classes “legal” or “illegal” as

(5) $$\left\{\begin{array}{c} \mathrm{Legal}\;\mathrm{step}\;(\mathrm{or}\;\mathrm{step}\;\mathrm{sequence})\;\mathrm{if}\;LOGC_{T}\left(\mathrm{or}\;\;\;LOGC_{T,S}\right)\leq LHE\\ \mathrm{Illegal}\;\mathrm{step}\;(\mathrm{or}\;\mathrm{step}\;\mathrm{sequence})\;\mathrm{if}\;LOGC_{T}\left(\mathrm{or}\;\;\;LOGC_{T,S}\right)>LHE \end{array}\right.$$

where LHE is the limit of human eye, set equal to 40 ms according to studies on human psycho-physiological limitations of vision and previous research on race walking [12]. This limit was also chosen in a similar classification proposed by Alvarez et al. [16].

##### Three-Level Classification

**Figure 6:**
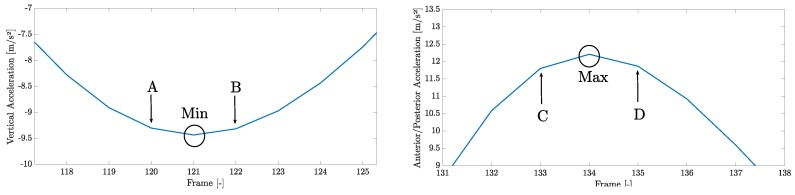
Acceleration data. Left, vertical accelerations close to the bottom point of the cycle; right, anteroposterior accelerations close to the maximum point of the cycle. On the abscissa axis, the time evaluated as the number of samples captured.

In this classification, the $LOGC_{T_{i}}$ values are expressed as confidence intervals, included between $LOGC_{T_{i,min}}$ and $LOGC_{T_{i,max}}$ values:

(6) $$LOGC_{T_{i,min}}=\left(t_{max_{i+1}\left(D\right)}+\frac{1}{f}\right)-\left(t_{min_{i}\left(A\right)}-\frac{1}{aSC_{i}^{2}+bSC_{i}}-\frac{1}{f}\right)$$

(7) $$LOGC_{T_{i,max}}=\left(t_{max_{i+1}\left(C\right)}-\frac{1}{f}\right)-\left(t_{min_{i}\left(B\right)}-\frac{1}{aSC_{i}^{2}+bSC_{i}}+\frac{1}{f}\right)$$

where *f* is the sample frequency of the inertial sensor. Equation (6) considers the points A and D in Figure 6, respectively, as the points of minimum vertical acceleration and maximum anteroposterior acceleration; Equation (7) indeed considers the points B and C in Figure 6, respectively, as the points of minimum vertical acceleration and maximum anteroposterior acceleration. From Equations (6) and (7), we obtain the same values but expressed for step sequences as

(8) $$LOGC_{T,S{,}_{min}}=\frac{1}{NS}\sum_{i=1}^{NS}LOGC_{T_{i,min}}$$

(9) $$LOGC_{T,S{,}_{max}}=\frac{1}{NS}\sum_{i=1}^{NS}LOGC_{T_{i,max}}$$

The three-levels classification assigns each step (or step sequence) to the classes “legal"”, “doubt”, or “illegal” as

(10) $$\left\{\begin{array}{c} \mathrm{Legal}\;\mathrm{step}\;(\mathrm{or}\;\mathrm{step}\;\mathrm{sequence})\;\mathrm{if}\;LOGC_{T_{i,min}}\left(\mathrm{or}\;LOGC_{T,S{,}_{min}}\right)<LHE\;\&\;LOGC_{T_{i,max}}\left(\mathrm{or}\;LOGC_{T,S{,}_{max}}\right)<LHE\\ \mathrm{Doubt}\;\mathrm{step}\;(\mathrm{or}\;\mathrm{step}\;\mathrm{sequence})\;\mathrm{if}\;LOGC_{T_{i,min}}\left(\mathrm{or}\;LOGC_{T,S{,}_{min}}\right)<LHE\;\&\;LOGC_{T_{i,max}}\left(\mathrm{or}\;LOGC_{T,S{,}_{max}}\right)>LHE\\ \mathrm{Illegal}\;\mathrm{step}\;(\mathrm{or}\;\mathrm{step}\;\mathrm{sequence})\;\mathrm{if}\;LOGC_{T_{i,min}}\left(\mathrm{or}\;LOGC_{T,S{,}_{min}}\right)>LHE\;\&\;LOGC_{T_{i,max}}\left(\mathrm{or}\;LOGC_{T,S{,}_{max}}\right)>LHE \end{array}\right.$$

**Figure 7:**
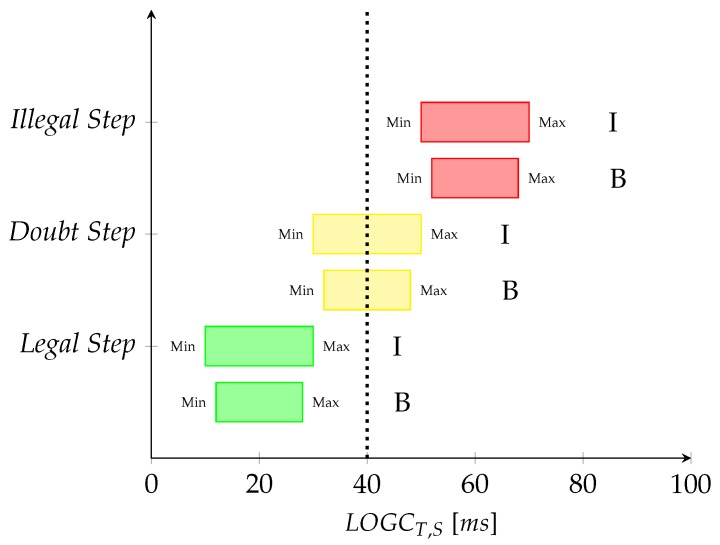
Three-levels step classification. On the *x*-axis, the values of $LOGC_{T,S}$ expressed in [ms]. On the *y*-axis, the classification of steps. Green, “legal” steps; yellow, “doubt” steps; red, “illegal” steps. I: inertial classification; B: benchmark classification (from the high-speed camera). The vertical dotted line represents the limit of human eye (LHE) value (fixed equal to 40 ms).

Figure 7 shows a graphical interpretation of the three-level classification. This picture also reports the comparison of the three-level classification using the inertial system (I) and the three-level classification using high-speed camera data, assumed as benchmark (B). The values in Equations (6)–(9) refer to the inertial data (I); indeed, the reference values of the benchmark classification are available in Section 2.4.2.

##### Fuzzy Classification

**Figure 8:**
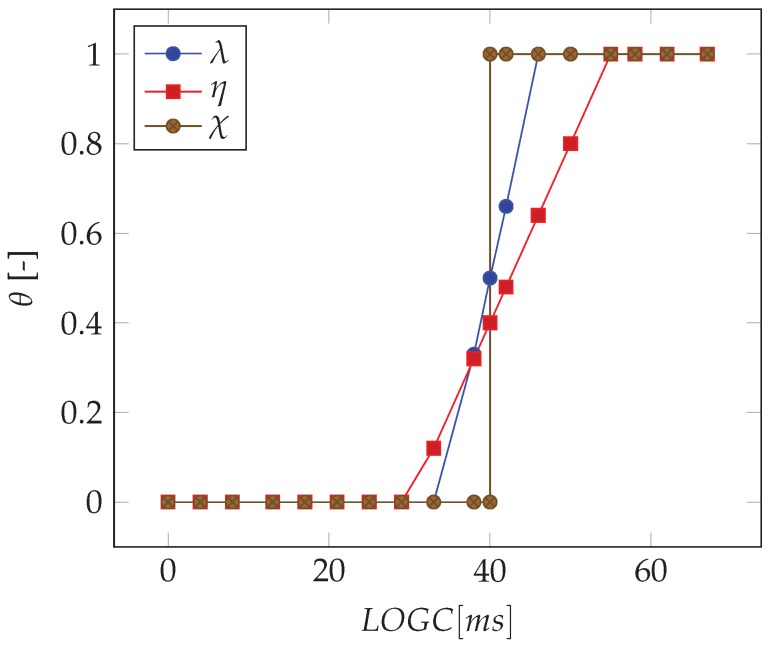
Membership functions for the inertial sensor (η) and the video benchmark system (λ). χ is the graphical representation of the binary classification. θ represents the output values of the functions.

This classification considers the “doubt case” by introducing a novel degree of uncertainty given by fuzzy numbers, which are used to manage step classification [17]. A fuzzy membership function is built to describe the response of the inertial system: it defines how each input space ($LOGC_{T}$ for a single step and $LOGC_{T,S}$ for a step sequence) is mapped to a membership value between 0 and 1 (output space). The membership value η is defined as follows.

(11) $$\begin{array}{c} \eta =\left\{\begin{array}{cc} \eta =0\;(\mathrm{Legal}\;\mathrm{step}\;\mathrm{or}\;\mathrm{step}\;\mathrm{sequence}) & LOGC_{T}\;(\mathrm{or}\;LOGC_{T,S})\;\leq (\mathrm{LHE}-\frac{2}{f})\\ 0<\eta <1\;(\mathrm{Doubt}\;\mathrm{step}\;\mathrm{or}\;\mathrm{step}\;\mathrm{sequence}) & (\mathrm{LHE}-\frac{2}{f})>\;LOGC_{T}\;(\mathrm{or}\;LOGC_{T,S})\;>(\mathrm{LHE}+\frac{2}{f})\\ \eta =1\;(\mathrm{Illegal}\;\mathrm{step}\;\mathrm{or}\;\mathrm{step}\;\mathrm{sequence}) & LOGC_{T}\;(\mathrm{or}\;LOGC_{T,S})\;\geq (\mathrm{LHE}+\frac{2}{f}) \end{array}\right. \end{array}$$

where LHE is again the limit of human eye, and *f* is the sample frequency of the inertial sensor. Figure 8 shows the membership function in Equation (11) compared with the binary classification method and the benchmark classification using video analysis (this is used for the validation, see, e.g., Section 2.4.2).

#### 2.2.3. Performance Parameters Assessment

The first performance parameters that we consider are step cadence (SC) and step length ratio (SLR). Indeed, the literature underlines that the ability of the best race walker is to achieve the optimal values of SC and SLR, with a legal LOGC timing [18]. For the *i*-th step, we have that the values for step cadence and step length ratio are

(12) $$SC_{i}=\frac{1}{t_{max_{i+1}}-t_{max_{i}}}$$

(13) $$SLR_{i}=\frac{v_{i,\mathrm{mean}}}{SC_{i}h}$$

where $v_{i,\mathrm{mean}}$ is the mean step speed and *h* is the athlete’s height. The values of SC and SLR for a step sequence are again the average values of a sequence of 30 steps as

(14) $$SC_{S}=\frac{1}{NS}\sum_{i=1}^{NS}SC_{i}$$

(15) $$SLR_{S}=\frac{1}{NS}\sum_{i=1}^{NS}SLR_{i}$$

Another interesting parameter is the “fluidity” of the race walker, which can be evaluated through the smoothness parameter (*S*) related to the anteroposterior acceleration. Indeed, this gives an estimation of the braking related to the anteroposterior direction. It can be evaluated using the model proposed in [19], for the *i*-th step and for the step sequence, as

(16) $$S_{i}=\sqrt{\frac{{\left(t_{max_{i+1}}-t_{max_{i}}\right)}^{5}}{{\left(\frac{1}{SC_{i}}v_{i,\mathrm{mean}}\right)}^{2}}\int_{t_{max_{i}}}^{t_{max_{i+1}}}j^{2}\left(t\right)dt}$$

(17) $$S_{S}=\frac{1}{NS}\sum_{i=1}^{NS}S_{i}$$

where j(t) is the jerk related to the anteroposterior acceleration.

### 2.3. Data Representation

In this subsection, we first report the development of five biomechanical indices customized for elite athletes and developed from the performance and infringements parameters. Then, we show their representation on a radar chart. Additional details on data processing and analysis are reported in Appendix A.

#### 2.3.1. Synthetic Biomechanical Indices

Here, we derive five normalized biomechanical indicators (δ, α, γ, ρ, and μ) starting from the previously defined parameters of infringements and performance related to step sequences ($LOGC_{T,S}$, $LOGC_{C,S}$, $SC_{S}$, $SLR_{S}$, $S_{S}$); indeed, the parameters evaluated for a sequence of steps are the most interesting from the regulation point of view. Furthermore, they are normalized such that they assume a value between 0 (best score) and 1 (worst score).

For the normalization of $LOGC_{T,S}$, we consider a parameter δ with two boundaries: δ=0, if LOGCT,S≤LHE−2f; δ=0.4, if $LOGC_{T,S}=LHE$. Then, we define a linear equation between δ = 0 and δ = 0.4, and we derive the following system of equations to describe δ.

(18) $$\begin{array}{c} \delta =\left\{\begin{array}{cc} \delta =0 & \;LOGC_{T,S}\leq (\mathrm{LHE}-\frac{2}{f})\\ \delta =\frac{1}{LHE-\frac{5}{f}}\cdot \left(LOGC_{T,S}-\left(LHE-\frac{2}{f}\right)\right) & \;(\mathrm{LHE}-\frac{2}{f})>LOGC_{T,S}>(\mathrm{LHE}+\frac{3}{f})\\ \delta =1 & \;LOGC_{T,S}\geq (\mathrm{LHE}+\frac{3}{f}) \end{array}\right. \end{array}$$

The parameter $LOGC_{C,S}$ does not need any normalization: indeed, according to Equation (4), it already results determined in a scale between 0 and 1; for it, we simply refer to α as

(19) $$\alpha =LOGC_{C,S}$$

For the normalization of $SC_{S}$ and $SLR_{S}$, we use the correlation equations derived in [15,18], based on elite competition data:

(20) $$SC_{S}=0.259v+2.253$$

(21) $$SLR_{S}=2.47v+32.73$$

where we indicate with *v* the race walker’s speed, expressed in [km/h] in Equation (20) and in [m/s] in Equation (21). Notice that $SC_{S}$ in Equation (20) is expressed as [steps/s], whereas $SLR_{S}$ in Equation (21) is without dimensions. From Equations (20) and (21), we extract the optimal values (called as $SC_{S,\gamma =0}$, $SLR_{S,\rho =0}$) and the ones which can be defined sufficient (called as $SC_{S,\gamma =0.4}$, $SLR_{S,\rho =0.4}$):

(22) $$\left\{\begin{array}{c} SC_{S,\gamma =0.4}\;\mathrm{with}\;v=v_{E}\\ SC_{S,\gamma =0}\;\mathrm{with}\;v=v_{R} \end{array}\right.$$

(23) $$\left\{\begin{array}{c} SLR_{S,\rho =0.4}\;\mathrm{with}\;v=v_{E}\\ SLR_{S,\rho =0}\;\mathrm{with}\;v=v_{R} \end{array}\right.$$

where $v_{E}$ is the speed of the qualifying standard time for the World Athletics World Championship of London 2017 for the 50 km men competition ($v_{E}$ = 12.20 km/h; 3.39 m/s), and $v_{R}$ is the speed of the world record for the 20 km men competition ($v_{R}$ = 15.76 km/h; 4.38 m/s). These values are chosen to cover all the speeds of interest. Consequently, using Equations (22)–( 23), we have $SC_{S,\gamma =0.4}$ = 3.130 steps/s and $SC_{S,\gamma =0}$ = 3.380 steps/s; $SLR_{S,\rho =0.4}$ = 62.8 and $SLR_{S,\rho =0}$ = 71.4. Then, we compute the normalized indices γ and ρ as

(24) $$\gamma =\left\{\begin{array}{cc} \gamma =1 & SC_{S}\leq (-1.5\cdot SC_{S,\gamma =0}+2.5\cdot SC_{S,\gamma =0.4})\\ \gamma =-0.4\frac{SC_{S}-SC_{S,\gamma =0.4}}{SC_{S,\gamma =0}-SC_{S,\gamma =0.4}}+0.4 & \\ \gamma =0 & SC_{S}\geq SC_{S,\gamma =0} \end{array}\right.$$

(25) $$\rho =\left\{\begin{array}{cc} \rho =1 & SLR_{S}\leq (-1.5\cdot SLR_{S,\rho =0}+2.5\cdot SLR_{S,\rho =0.4})\\ \rho =-0.4\frac{SLR_{S}-SLR_{S,\rho =0.4}}{SLR_{S,\rho =0}-SLR_{S,\rho =0.4}}+0.4 & \\ \rho =0 & SLR_{S}\geq SLR_{S,\rho =0} \end{array}\right.$$

Finally, the smoothness parameter $S_{S}$ is normalized considering the following boundary values: $S_{S,min}$ = 1 (ideal smoothness); $S_{S,max}$ = 10 (worst possible value for smoothness). We, therefore, define the parameter μ as

(26) $$\mu =\frac{S_{S}-S_{S,min}}{S_{S,max}-S_{S,min}}$$

#### 2.3.2. Radar Chart Representation

**Figure 9:**
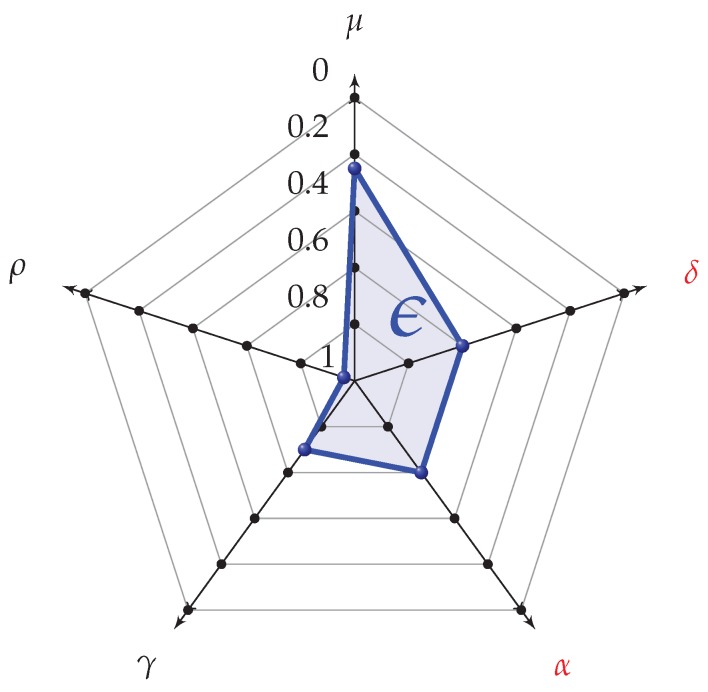
Radar chart representation of the five biomechanical indices. The red indices on the right (α and δ) are related to infringements, and the black indices on the left (μ, ρ γ) are related to performance. The blue opacity area (ϵ) represents the synthetic index.

The biomechanical indices derived in Section 2.3.1 are graphically plotted on a radar chart, which offers a synthetic and intuitive representation of infringements and performance (see, e.g., Figure 9). The calculation of the polygon area (*A*, blue area in Figure 9) allows obtaining of an overall synthetic index (referred to as ϵ) for the evaluation of the overall gesture of the athlete. Indeed, this index allows to consider the infringement within the analysis of performance. This index is expressed as

(27) $$\epsilon =\frac{A}{A_{Max}}$$

where $A_{Max}$ is the maximum achievable area (area of a regular pentagon with unitary radius). Furthermore, we establish the minimum condition to ensure an admissible level of correct technique, and we define the best admissible value $\epsilon_{opt}$ as

(28) $$\left\{\begin{array}{c} \epsilon_{opt}=\frac{A}{A_{Max}}\\ \delta \leq 0.4\\ \alpha \leq 0.4 \end{array}\right.$$

where we have considered the maximum tolerable values for the infringements parameters (α and δ equal to 0.4).

### 2.4. Validation Strategy

In this section we present the procedures used to validate the main contributions of the methodology (i) LOGC timing assessment, (ii) LOGC classification, and (iii) biomechanical indices and radar chart representation. As our method starts from the approach proposed by Lee et al. in [9], we compare most of results of the current paper with respect to the previous Lee’s method. For this comparison, as the latter method considers steps instead of step sequences, we also validate the classification of LOGC with respect to single steps.

#### 2.4.1. LOGC Timing

We validate our proposal for estimation of $LOGC_{T}$ with respect to (1) benchmark values obtained by an high-speed camera system (called as $LOGC_{B}$) and (2) values obtained by the method proposed by Lee et al. in [9], which consider a fixed value for the threshold *E* in Equation (3) (we call these values as $LOGC_{T,Lee}$). For (1), the video motion analysis is performed using the Kinovea© software (Joan Charmant&Contrib.). We compute $LOGC_{B}$ as the time interval between the frame corresponding to TOE to the following frame of HSE. Thus, we evaluate (i) the difference in the detection of LOGC events between inertial and high-speed camera systems and (ii) the timing difference between $LOGC_{T}$ and $LOGC_{B}$ and between $LOGC_{T,Lee}$ and $LOGC_{B}$ in terms of mean and standard deviation values.

#### 2.4.2. LOCG Classification

We evaluate the three inertial-based classification strategies of this work with respect to the same classifications obtained from video analysis, assumed as reference. For step sequence classification, the loss of ground contact timing assessed by high-speed camera is computed as

(29) $$LOGC_{B,S}=\frac{1}{NS}\sum_{i=1}^{NS}LOGC_{B_{i}}$$

First, from high-speed camera data and from inertial sensor data, all steps are classified as “legal” or “illegal” according to the binary classification, and the confusion matrix for each trial is obtained. Assuming as true the results from the classification based on the high-speed camera, the false alarm rate, the miss alarm rate, the accuracy, the true positive rate (TPR), and the false positive rate (FPR) are derived. The accuracy value for the binary classification indicates the ability of the system in the discrimination between legal and illegal steps. From the TPR and FPR, we construct the ROC graph [20,21] for comparing the performances of the two classifiers using the Lee’s and the proposed methods.

Second, we proceed with the validation of the three-levels classification. Therefore, for the *i*-th step, we define the following boundaries of the benchmark values $LOGC_{B_{i,max}}$ and $LOGC_{B_{i,min}}$ as

(30) $$LOGC_{B_{i,max}}=\frac{1}{FR}-\left(FN_{D_{i}}-FN_{A_{i}}\right)$$

(31) $$LOGC_{B_{i,min}}=\frac{1}{FR}-\left(FN_{C_{i}}-FN_{B_{i}}\right)=LOGC_{B,i_{max}}-\frac{2}{FR}$$

For the step sequence classification, we use indeed the following boundary values.

(32) $$LOGC_{B,S_{max}}=\frac{1}{NS}\sum_{i=1}^{NS}LOGC_{B_{i,max}}$$

(33) $$LOGC_{B,S_{min}}=\frac{1}{NS}\sum_{i=1}^{NS}LOGC_{B_{i,min}}$$

**Figure 10:**
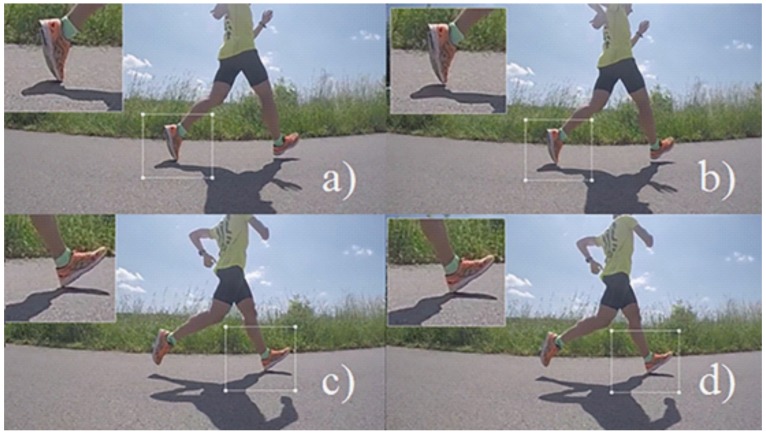
Photo sequence with magnifying glass focused on the left foot (near the TOE, upper pictures) and on the right foot (near the HSE, bottom pictures). (**a**) Last image with visible ground contact of left foot. (**b**) First image without the last visible ground contact of left foot. (**c**) Last image without visible ground contact of right foot; (**d**) First image with visible ground contact of right foot.

In Equations (30) and (31), FR is the frame rate of the high-speed camera expressed as [frames/s]; $FN_{A}$ is the frame number corresponding to the last frame in which the contact with the ground is visible (see Figure 10a); $FN_{B}$ is the frame number corresponding to the frame following the last contact with the ground (see Figure 10b); $FN_{C}$ is the frame number corresponding to the last frame before the contact with the ground is visible (see Figure 10c); $FN_{D}$ is the frame number corresponding to the first frame in which the contact with the ground is visible (see Figure 10d). Then, the TPR and Predict Positive Value (PPV) values of the multi-class confusion matrices (related to Lee’s and our proposed approach) are plotted on the precision–recall curve and the area under the curve (AUC) is computed for comparison of the two classifiers.

Third, for validation of the fuzzy classification, we define λ($LOGC_{B,S}$) as the membership function for the fuzzy set $LOGC_{B,S}$. This is built by expressing the high-speed camera data as confidence intervals whose maximum and minimal values are given by Equations (32) and (33). The core part includes all $LOGC_{B,S}$ values with a confidence interval over LHE; the boundary includes all $LOGC_{B,S}$ with a confidence interval crossing LHE; all other cases are related to confidence intervals under LHE. The plot of λ($LOGC_{B,S}$) is shown in Figure 8, and its expression is given by

(34) $$\begin{array}{c} \lambda \left(LOGC_{B,S}\right)=\left\{\begin{array}{c} \lambda =0\;(\mathrm{Legal}\;\mathrm{step}\;\mathrm{sequence})\;LOGC_{B,S}\;\leq \;(\mathrm{LHE}\;-\frac{2}{f})\\ 0<\lambda <1\;(\mathrm{Doubt}\;\mathrm{step}\;\mathrm{sequence})\;(\mathrm{LHE}\;-\;\frac{2}{f})\;>\;LOGC_{B,S}\;>\;(\mathrm{LHE}\;+\;\frac{2}{f})\\ \lambda =1\;(\mathrm{Illegal}\;\mathrm{step}\;\mathrm{sequence})\;LOGC_{B,S}\;\geq \;(\mathrm{LHE}\;+\;\frac{2}{f}) \end{array}\right. \end{array}$$

In addition, we define σ equal to the difference between η in Equation (11) and λ in Equation (34). This parameter allows to measure the distance between the inertial and camera systems. Then, we define the following criterion for declaring a correct identification of the steps.

(35) $$\left\{\begin{array}{cc} -0.5<\sigma <0.5 & \mathrm{Acceptable}\;\mathrm{Classification}\;\left(\mathrm{AC}\right)\\ \sigma \leq -0.5;\sigma \geq 0.5 & \mathrm{Wrong}\;\mathrm{Classification}\;\left(\mathrm{WC}\right) \end{array}\right.$$

From Equation (35), the percentage of acceptable classification τ is defined as

(36) $$\begin{array}{c} \tau =\frac{AC}{AC+WC} \end{array}$$

The index in Equation (36) represents the ability of the system to give an acceptable output (close to the benchmark system). The computation of Equation (36) is carried out for both the proposed method and for the Lee’s one.

#### 2.4.3. Synthetic Biomechanical Indices and Radar Chart Representation

The performance and infringement biomechanical indices (derived in Section 2.3.1) are screened (i) for normality of distribution, using the normality test of Kolmogorov–Smirnov, and (ii) for homogeneity of variances, using the Levene’s test. The magnitude of differences, also called effect sizes (ES), for each parameter and for the related key performance index at different speeds, are calculated according to Hedges’ g value and interpreted as trivial (ES ≤ 0.25), small (>0.25 and <0.5), moderate (≥0.5 and <1.0), and large (≥1.0), following the scale proposed by Fröhlich [22] for highly trained participants. Finally, to measure the weight of the key performance indices (δ, α, γ, ρ, μ) on the race walking overall index (ϵ), we introduce the $\kappa_{i}$ indices as

(37) $$\kappa_{i}=\frac{H_{i}^{m,n}}{\sum_{i}H_{i}^{m,n}}$$

where $H_{i}$ represents the Hedges’ g value for a generic key performance index *i* evaluated between the groups at minimum speed (*m*) and maximum speed (*n*).

## 3. Results and Discussions

In this section, we present the results of the main contributions of this work: (i) inertial-based calculation of $LOGC_{T}$ with variable threshold *E* (presented in Section 2.2.1), (ii) step classification methods (presented in Section 2.2.2), and (iii) biomechanical indices and radar chart representation (presented in Section 2.3). These are validated using the strategies proposed in Section 2.4.

### 3.1. LOGC Timing

We compute the $LOGC_{T}$ from nine tests of three different athletes (two men: one specialized on 20 km, one on 50 km; one woman specialized on 20 km). These tests are chosen for the following reasons; (i) to cover a full range of speeds of an elite race walker (from 12.0 km to 15.5 km/h); (ii) to analyze specialists from all main competitions. We choose five different speeds to validate the results for all the athlete’s range of speeds. For each race walking test, excluding the initial acceleration phase of the athlete (10 s), 180 consecutive steps are considered (corresponding to six step sequences). A total amount of 1620 athlete’s steps are thus evaluated. Table 4 reports the LOGC timing values computed with: high-speed camera ($LOGC_{B}$), inertial system with Lee’s method ($LOGC_{T,Lee}$) and inertial system with the proposed method ($LOGC_{T}$), as well as the number of events of LOGC detected with the three approaches. Furthermore, this table also reports the mean differences between the estimation of LOGC timing using the Lee’s approach and high-speed camera ($MD_{L}$) and between the proposed approach and high-speed camera ($MD_{P}$).

The analysis of Table 4 underlines that the inertial system with the proposed approach allows to correctly identify 1606 LOGC events, with a 99% of correct detection rate. Only fourteen errors happen: eleven LOGC are classified as double supports (i.e., no loss of ground contact has occurred) and three double supports are indeed classified as LOGC. Similar scores are obtained with the Lee’s approach (1609 correct detection of LOGC events). Notice that in [9], the authors have reached an accuracy of 91% in the correct detection of LOGC events, by using the Lee’s method. Regarding the LOGC timing duration, we can see from the Table 4 that these durations are directly proportional to the test speed: this is in agreement with previous works [15]. The values of $MD_{L}$ and $MD_{P}$ show that with the proposed approach, we reduce the mean difference of correct evaluation of LOGC timing, compared with respect to the benchmark system; therefore, $LOGC_{T}$ values and closer to $LOGC_{B}$ than $LOGC_{T,Lee}$. We also appreciate that the mean values of $MD_{P}$ are below 0.02 s, which is the mean error reached by Lee et al. in [9].

### 3.2. LOGC Classification

Here, we report the results of LOGC classification for binary classifier, for step and step sequences; three-levels classifier, only for step sequences; and fuzzy classifier, for step and step sequences.

Table 5 reports the results of step binary classification, with respect to the following statistical parameters calculated for the Lee’s and proposed approaches: false alarm, miss alarm, true positive rate (TPR), false positive rate (FPR), and accuracy. These values are calculated for different speeds, and they are obtained from the confusion matrices in Table A1 and Table A2, reported in Appendix C. The most important value of this table is the accuracy. This parameter still show a decreasing trend with speed except at the highest speed; however, we can notice that the accuracy values of our proposed method (P) are higher than the ones obtained with the Lee’s method (Lee). The average increasing of accuracy is equal to +14% (from +1% for 15.5 km/h till +32% for 14.5 km/h). For all speeds, the accuracy values of our method all overcome the threshold of 70%, with a mean accuracy value of 81%. The false alarm rate also shows an improvement; indeed, it decreases from 4% at 12.0 km/h till 39% at 14.5%, remaining equal only at 15.5 km/h. Only the miss alarm rate is worsened.

Table 6 indeed reports the results of binary classification on step sequences, by computing the same statistical parameters of the previous table for the Lee’s and proposed approaches. Again, Table 6 is based on the confusion matrix in Table A3, reported in Appendix C. From the analysis of the step sequence binary classification (Table 6), we can see that the proposed method allows achieving a better accuracy (87%, with an increasing of 17%) than the Lee’s method. Again, also for the step sequence, the proposed method shows better performances with respect to false alarm (from 37% to 14%), whereas the worsening trend of miss alarm is confirmed (from 0% to 9%).

The outputs of binary step classification and step sequence classification underlines how the proposed approach shows (i) overall enhanced performances in comparison with Lee’s approach, especially for accuracy, and (ii) pattern of evaluation similar to those of real judgments, but with better performances. For giving a better understanding of these values, in [23], an outdoor experiment is reported where the judges’ assessment is compared to a camera evaluation. Analyzing these data with our binary method of classification for step sequence, the judges reached the following accuracy score; 73%, 68%, and 54% (mean value of 65%), which is far below the accuracy values that we have reached in this paper. Furthermore, we have underlined that the values of accuracy decrease in the range of speeds between 13.0 km/h and 14.0 km/h (respectively, they are equal to 73% and 70%, see Table 5); at these speeds, the LOGC timing values are between 40 ms and 45 ms. A recent study carried out by Hanley et al. [24] underlines that LOGC timing values between 40 ms and 45 ms are usually detected by no more than 37% of judges. In the paper [24], it is also reported than for LOGC timing values below 33 ms (typical of speeds below 13.0 km/h), 12.5% of judges detect a non-visible LOGC (that according to LHE can be considered as a false alarm): in our work, at the speed of 12.0 km/h, we have reached 8% of false alarm, which is better than 12.5%. The better accuracy is obviously reached at higher speeds: for example, judges are able to reach 85% of accuracy with LOGC equal to 60 ms [24], whereas our approach in similar LOGC timing conditions (15.5 km/h, where LOGC timing values are above 50 ms) reaches 94% of accuracy. We believe that these accuracy values can be further improve by taking into consideration also the race walker’s anthropocentric characteristics (as the height of CoM) in the computation of *E* in Equation (3).

**Figure 11:**
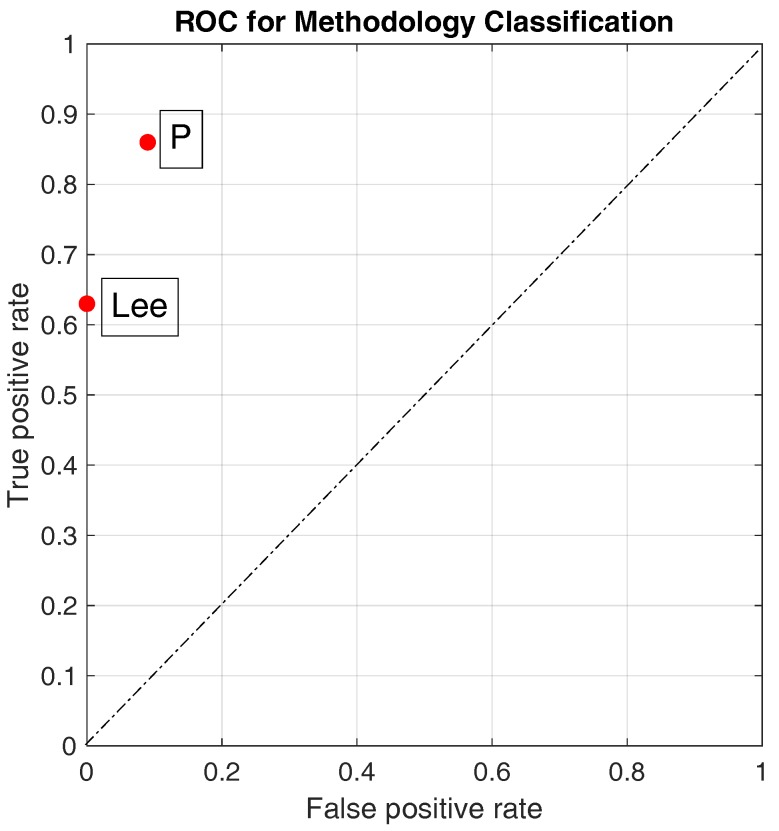
ROC graph showing the two approaches for binary classification. (Lee): Lee’s approach; (P): Proposed approach.

To complete the comparison between the Lee’s and proposed approaches for binary classification, we plot, in the ROC space of Figure 11, the two points corresponding to the pairs (FPR and TPR), see Table 6. As the two points are above the diagonal, both the approaches show good a classification. However, as the distance of (P) from the point (0,1), which indicates the perfect classifier, is lower than the distance of (Lee) from the point (0,1), the proposed classifier shows better performances.

Then, data corresponding to the 54 step sequences (1620 steps) have been analyzed through the three-level classification on step sequences. Table 7 reports the statistical parameters TPR (true positive rate) and positive PPV (predict positive value) for “legal”, “doubt”, and “illegal” step sequences, as well as the accuracy values of the Lee’s and proposed methods. Again, this table is based on the multi-class confusion matrices in Table A4, reported in Appendix C. Table 7 shows that the proposed approach outperform the Lee’s approach with respect to accuracy, true positive rate for legal steps, and predict positive value for doubt steps. The accuracy values of the three-level classification appears to be worse than the corresponding values with two levels (i.e., binary classification). This happens because many legal sequences of steps are now classified as “doubt” cases (as shown by $PPV_{L}$). However, this error in competitions is not a problem because it could represent only a warning for an athlete (not a disqualification).

**Figure 12:**
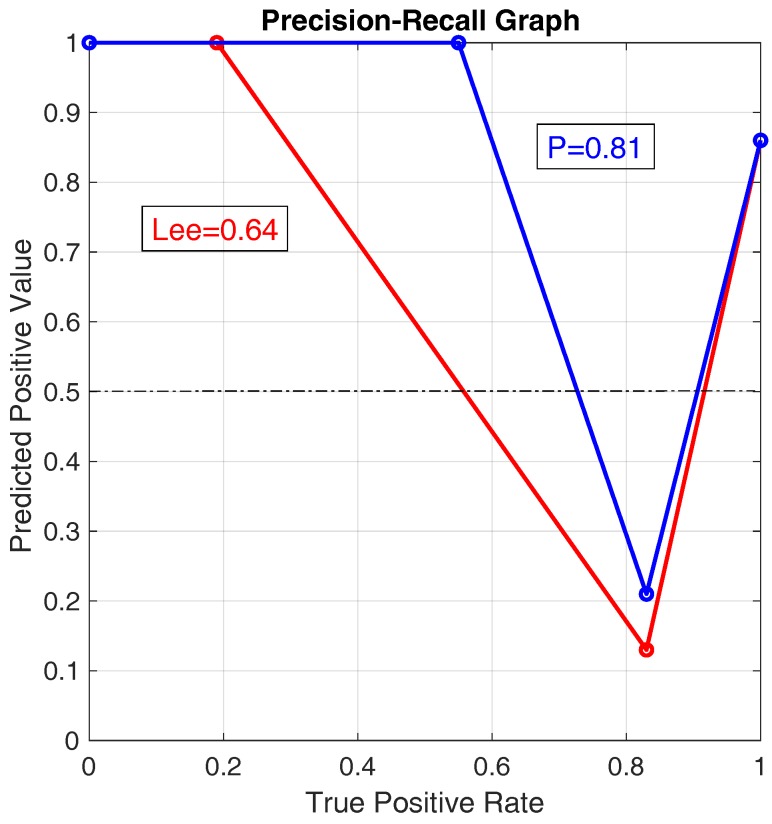
Precision recall graph for the two approaches with three-levels step classification. (Lee): Lee’s approach; (P): Proposed approach.

Furthermore, for the three-level classification, we compare the results of both approaches in the precision recall graph shown in Figure 12. In this figure, we plot the points corresponding to the couples (TPR, PPV) for “legal”, “doubt”, and “illegal” step sequences as ($TPR_{L}$,$PPV_{L}$), ($TPR_{D}$,$PPV_{D}$), ($TPR_{I}$,$PPV_{I}$), for both approaches. As the $TPR_{L}$ values are different from zero for the two approaches, we add an additional point at (0,1). Then, the AUC values are obtained for the two curves. A value of AUC equal to 1.0 corresponds to an ideal classifier, while a value of AUC equal to 0.5 corresponds to a classifier with random performance level. The plot clearly shows that the classifier (P) outperforms the classifier (Lee), as it has an higher value of AUC (0.81 compared to 0.64).

Finally, the 1620 steps under investigation have been analyzed through the fuzzy classification method (see Section 2.4.2) using the two membership functions of the high-speed camera (λ in Equation (34)) and the inertial system (η in Equation (11)). Table 8 shows the results of the fuzzy classification on steps and on step sequences for the two approaches, in terms of the parameter τ defined in Equation (36). The correct classification shows a decreasing trend with speeds until the minimum value reached at 14.5 km/h; then, the trend changes. However, also the τ value confirms an improvement with the proposed approach at each speed (the mean value of $\tau_{P}$ is equal to 84%, which is +21% greater than $\tau_{Lee}$). The index $\tau_{P}$ is greater than $\tau_{Lee}$ also for step sequences, with a value higher than 90%.

In summary, to identify the best approach for LOGC classification, we have reported the following.

- Accuracy values for the binary classification (Table 5 for steps and Table 6 for step sequences);
- accuracy values for the three-level classification (Table 7 for step sequences);
- ROC and Precision-Recall classifier performances (Figure 11 for step sequence binary classification and Figure 12 for step sequence three-levels classification);
- percentage of acceptable classification values for the fuzzy classification (Table 8 for both steps and step sequences).

**Figure 13:**
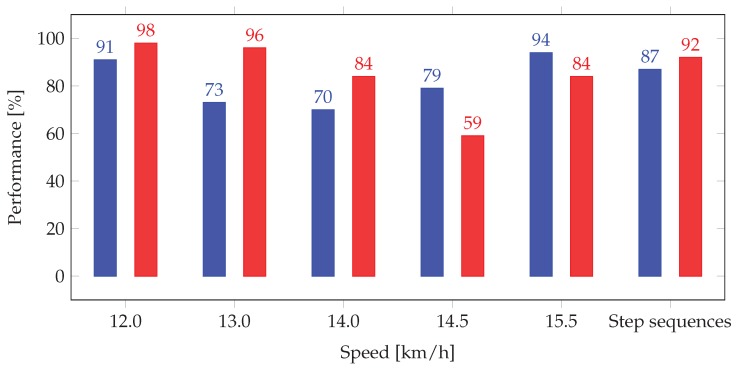
Comparison between binary and fuzzy classifications at different speeds. Blue bars, performance index of the binary classification (i.e., accuracy); red bars, performance index of the fuzzy classification (i.e., percentage of acceptable classification).

The bar plot in Figure 13 shows the comparison between the performances of binary and fuzzy classifications, for both step and step sequences. The measure for the binary classification is the accuracy value, while the measure for the fuzzy classification is the percentage of acceptable classification. The proposed approach guarantees good values for both the analyzed classifications, with 87% of accuracy in the discrimination of step sequences and 92% of acceptable differences with respect to the benchmark system.

### 3.3. Synthetic Biomechanical Indices and Radar Chart Representation

Here, we analyze the overall data collected by the nine race walkers, related to four speeds between 12.0 km/h and 14.5 km/h. A total amount of 36 tests (864 sequences of steps, 25,920 steps) were evaluated for all the athletes. Table 9 and Table 10 report the performance/infringement parameters and the related normalized biomechanical indices.

**Figure 14:**
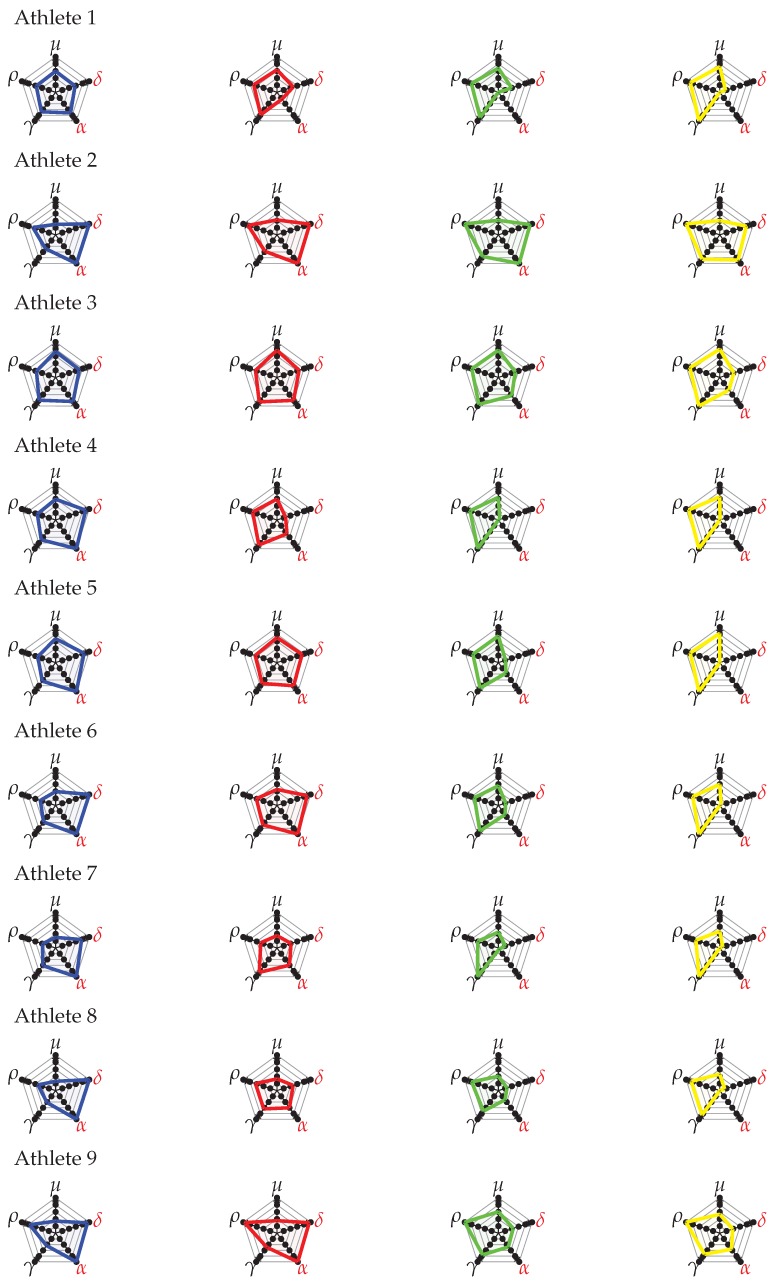
Radar charts for the experimental tests, for all the athletes involved in the study. The color lines graphically show the trend of the indices at different speeds (blue, 12.0 km/h; red, 13.0 km/h; green, 14.0 km/h; yellow, 14.5 km/h). The wider the area is, the better the gesture is.

The analysis for nine athletes confirms the key points shown in the evaluation of LOGC timing and step classification: (i) the increasing trend of $LOGC_{T,S}$ and δ values with the speed (and also of $LOGC_{C,S}$ and α); (ii) for speeds slower than 13 km/h, the mean $LOGC_{T,S}$ of step sequences are under LHE (as previously fixed equal to 40 ms), and only few sequences have $LOGC_{T,S}$ greater than LHE ($LOGC_{C,S}$ and α value close to 0). In addition, also the $SC_{S}$ and $SLR_{S}$ values (and the related indices γ and ρ) increase with speed; this is in accordance with the literature [15,18]. Indeed, with increasing step frequencies, the smoothness improves (decreasing values for $S_{S}$ and μ): this is again in accordance with the literature [15,18]. Figure 14 reports the biomechanical indices represented on radar charts, for all the nine athletes. From the performance analysis point of view, the radar chart allows to understand strong and critical points that characterize the gesture of the race walker. For example, the radar charts underline how Athlete 2 and Athlete 9 have step length values (ρ) better than step cadence values (γ); therefore, step length values represent their strong point. Indeed, Athletes 5, 6, and 7 have the strongest technical feature in step cadence. Regarding the development of the biomechanical indices, a further improvement could be done by using different normalization strategies customized for the main types of race competitions (men’s and women’s 20 and 50 km): we expect that this could improve the analysis of the athlete’s gesture for specific races. As a matter of fact, the authors in [24] underline the differences in values of SC, SLR among women and men elite race walkers. Finally, the index ϵ allows to individuate the speed where the graph area has the maximum value. Indeed, this value can suggest the speeds of the best compromise to achieve at the same time the optimal $SLR_{S}$ and $SC_{S}$ values, while ensuring an acceptable level of correct technique ($\epsilon_{opt}$). The analysis of Table 10 underlines that this speed varies among athletes with values between 12 and 14 km/h; this is again in accordance with the work in [24], where the authors have suggested the value of 14 km/h for men and 13 km/h for women, as limits for avoiding visible loss of ground contact.

**Figure 15:**
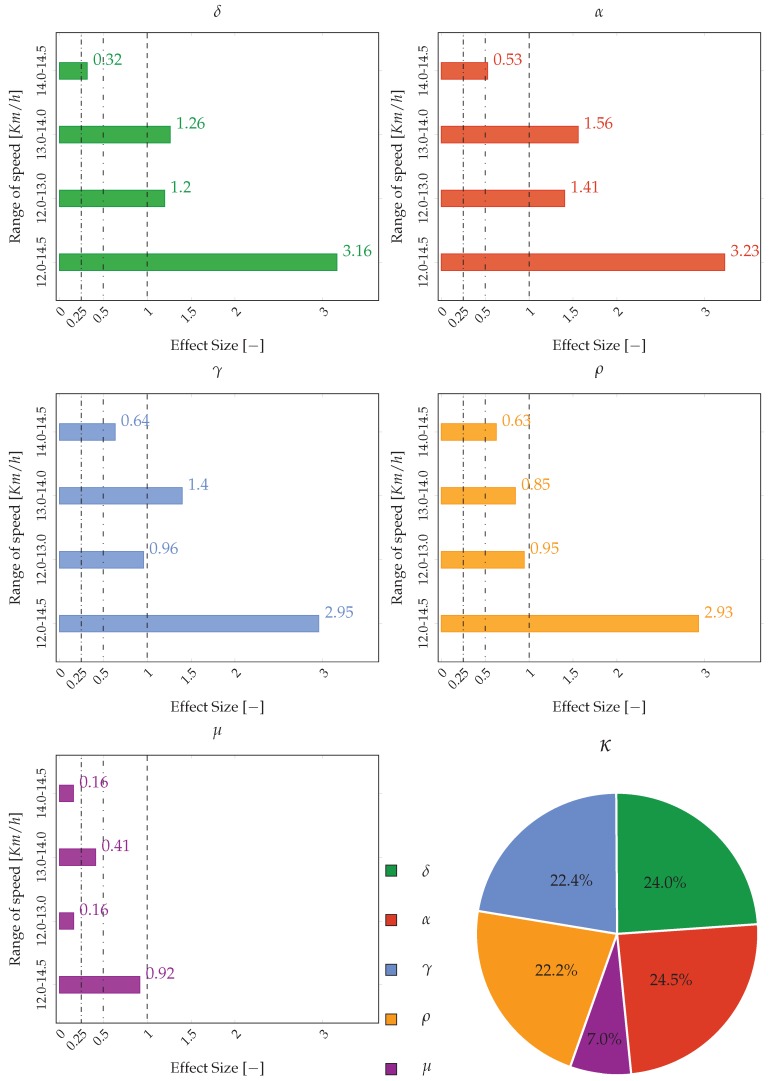
ES bar plots. Dash-dotted lines, trivial values of ES; loosely dash dotted lines, small values of ES; dashed lines, moderate values of ES. Only the the ES values over the lines of moderate values can be considered large values. The pie graph in the bottom right shows the percentage values of κ for all the indices.

Figure 15 reports the ES analysis. The infringement indices show large values of ES for these range of speeds: 12.0–13.0 km/h and 13.0–14.0 km/h. Indeed, the range of 14.0 to 14.5 km/h shows smaller values of ES (small for δ and moderate for α). The performance indices γ and ρ always have moderate ES values, with similar trends (except for γ, which has large ES values between 13 and 14 km/h, with respect to ρ). Moreover, again there is a reduction of ES in the last range of speeds (14.0–14.5 km/h), characterized by a smaller gain of speed. The third performance index (μ) shows trivial ES values. In the case of smoothness, to analyze a possible more significant variance, an additional investigation could be done on smoothness rotation indices (related with the vertical angular speed). It is important to notice that the reduction of ES value in the last range of speeds, 14.0–14.5 km/h, underlined both in the infringement parameters (δ and α) and in the performance ones (ρ and γ), is also related with the reduction of speed incremental gain (from 1.0 to 0.5 km/h). Furthermore, Figure 15 reports a pie graph with the calculation of κ indices according to (37). We can observe how, even if the infringement indices are fewer in number than the performance ones (2 compared to 3, respectively), their weight represents almost 50% of the total. This demonstrates their important role in the definition of the total area ϵ, as well as a good balance between performance and infringement indices contribution in the radar chart structure. In addition, a study based on ranking the relative importance of the indices from end-user’s perspective [25], could be useful to improve the radar chart evaluation.

## 4. Conclusions

In this paper, we have described the development of IART, a novel wearable inertial system for automatic detection of infringements and analysis of sports performance in race walking. We have derived five biomechanical indicators, normalized and customized for elite athletes, and we have represented them on a radar chart, for an intuitive evaluation of the athlete’s overall gesture. Then, we have validated the proposed indices in field environments with nine elite athletes. The results of the experimental validation have confirmed that (i) the proposed system represents a reliable and valuable tool to estimate the LOGC timing and identify legal and illegal steps in race walking, (ii) the IART system outperforms the score performance of a judges’ evaluation, although there is not an exact limit for LOGC detection, and (iii) the radar chart representation offers the possibility to build a customized profile of the race walkers useful for improvement of training strategies. As a matter of fact, the radar charts can highlight strengths and weakness of the athlete’s technique and they can suggest the speeds of the optimal compromise to achieve the best performances with acceptable values of parameters related to infringements.

## Figures and Tables

**Table 1:** Mean and standard deviation (SD) values among the participants related to: personal best on 20 km (PB), age, stature, and experience.

Statistics	PB [km/h]	Age [Years]	Stature [cm]	Experience [Years]
Mean	13.8	25.3	174.5	11.7
SD	0.7	4.7	4.0	5.5

**Table 2:** Technical features of the inertial sensor G-Sensor2 (BTS Bioengineering, Milan, Italy).

Technical Features	Accelerometer	Gyroscope
Dynamic Range	±8 g	±300 dps
Sample Frequency	200 Hz (1/5 ms)	
Noise	0.35 × 10^−3^ g/$\sqrt{\mathrm{Hz}}$	0.018 dps/$\sqrt{\mathrm{Hz}}$
Bandwidth	400 Hz	140 Hz
Battery autonomy	24 h	
Dimension	78 × 48 × 20 mm	
Weight	62 g	

**Table 3:** Parameters of the quadratic models.

Model	R-Squared	R-Squared adj	S
Quadratic LAR	98.91	98.87	0.008655
Quadratic	60.15	60.10	0.008008

**Table 4:** Detected events of LOGC and LOGC timing values, calculated according to inertial analysis (Lee’s and Proposed approach) and high-speed camera (benchmark). The benchmark values are ${\mathrm{Events}}_{B}$ and $LOGC_{B}$; the values calculated according to Lee’s method are ${\mathrm{Events}}_{Lee}$ and $LOGC_{T,Lee}$; the values calculated according to our proposed method are Events and $LOGC_{T}$. $MD_{L}$ and $MD_{P}$ refer, respectively, to the mean difference in LOGC estimation between the Lee’s approach and video analysis, and between our proposed approach and the video analysis.

Speed	${\mathrm{Events}}_{B}$	${\mathrm{Events}}_{Lee}$	Events	*LOGC_B_*	*LOGC_T,Lee_*	*LOGC_T_*	*MD_L_*	*MD_P_*
[km/h]	[-]	[-]	[-]	[ms]	[ms]	[ms]	[ms]	[ms]
12.0	536	540	529	17 ± 8	35 ± 10	20 ± 10	20 ± 10	0 ± 10
13.0	360	355	356	29 ± 8	45 ± 10	35 ± 10	15 ± 15	5 ± 10
14.0	360	360	360	37 ± 8	50 ± 10	40 ± 10	15 ± 15	5 ± 10
14.5	180	178	180	41 ± 8	50 ± 15	45 ± 10	5 ± 20	0 ± 10
15.5	180	180	180	50 ± 8	55 ± 5	65 ± 10	5 ± 10	15 ± 5

**Table 5:** Results of binary classification on step, with respect to the statistical parameters (false alarm, miss alarm, TPR, FPR, and accuracy) for the two approaches: Lee (Lee) and proposed (P), at different speeds. The best values for each row and for each pair of columns are highlighted with the symbol *.

	12.0	12.0	13.0	13.0	14.0	14.0	14.5	14.5	15.5	15.5
Statistics	km/h	km/h	km/h	km/h	km/h	km/h	km/h	km/h	km/h	km/h
	(Lee)	(P)	(Lee)	(P)	(Lee)	(P)	(Lee)	(P)	(Lee)	(P)
false alarm	12%	8% *	45%	24% *	64%	33% *	53%	14% *	100% *	100% *
miss alarm	55% *	64%	13% *	52%	13% *	22%	53% *	55%	1%	0% *
TPR	88%	92% *	55%	76% *	36%	66% *	47%	86% *	0% *	0% *
FPR	54% *	64%	13% *	52%	12% *	22%	53% *	54%	1%	0% *
accuracy	88%	91% *	58%	73% *	51%	70% *	47%	79% *	93%	94% *

**Table 6:** Results of binary classification on step sequence, with respect to the statistical parameters (false alarm, miss alarm, TPR, FPR, and accuracy) for the two approaches: Lee (Lee) and proposed (P), at different speeds. The best values for each row are highlighted with the symbol *. The Δ value represents the difference of each statistical parameter between the two methods (P-Lee).

Statistics	Step Sequences (Lee) [%]	Step Sequences (P) [%]	Δ [%]
false alarm	37	14 *	−23
miss alarm	0 *	9	+9
TPR	63	86 *	+23
FPR	0 *	9	+9
accuracy	70	87 *	+17

**Table 7:** Results of three-level classification on step sequences, with respect to the statistical parameters ($TPR_{L}$, $TPR_{D}$ and $TPR_{I}$: true positive rate for “legal”, “doubt”, and “illegal” step sequences; $PPV_{L}$, $PPV_{D}$ and $PPV_{I}$: predict positive value for “legal”, “doubt”, and “illegal” step sequences) for Lee’s (Lee) and proposed (P) approaches. The best values for each row are highlighted with the symbol *. The Δ value represents the difference of each statistical parameter between the two approaches (P-Lee).

Statistics	*TPR_L_* [%]	*TPR_D_* [%]	*TPR_I_* [%]	*PPV_L_* [%]	*PPV_D_* [%]	*PPV_I_* [%]	Accuracy [%]
(Lee)	19	83 *	100 *	100 *	13	86 *	35
(P)	55 *	83 *	100 *	100 *	21 *	86 *	63 *
Δ	+36	0	0	0	+8	0	+28

**Table 8:** Results of fuzzy classification on steps and step sequences, using the percentage of acceptable classification τ parameter for the two approaches: Lee’s (Lee) and proposed (P). The best value of τ for each speed are highlighted with *. Δ represents the difference between $\tau_{P}$ and $\tau_{Lee}$.

Speed [km/h]	$\tau_{Lee}$ [%]	$\tau_{P}$ [%]	Δ [%]
12.0	89	98 *	+9
13.0	56	96 *	+40
14.0	43	84 *	+41
14.5	46	59 *	+13
15.5	83	84 *	+1
Step sequences	73	92 *	+19

**Table 9:** Infringements ($LOGC_{T,S}$, $LOGC_{C,S}$) and performance ($SLR_{S}$, $SC_{S}$, $S_{S}$) parameters evaluated for 864 step sequences, corresponding to 25,920 steps (mean±SD).

Speed [km/h]	*LOGC_T,S_* [ms]	*LOGC_C,S_* [-]	*SC_S_* [Steps/s]	*SLR_S_* [%]	*S_S_* [-]
12.0	21 ± 7	0.08 ± 0.09	3.10 ± 0.07	61.9 ± 2.8	6.28 ± 1.40
13.0	34 ± 7	0.34 ± 0.25	3.20 ± 0.08	64.6 ± 3.2	5.39 ± 1.44
14.0	45 ± 8	0.63 ± 0.26	3.29 ± 0.07	67.3 ± 3.1	4.65 ± 1.12
14.5	51 ± 9	0.75 ± 0.26	3.34 ± 0.07	69.3 ± 3.3	4.44 ± 1.19

**Table 10:** Infringements (δ and α) and performance (γ, ρ, and μ) normalized biomechanical indices evaluated for 864 step sequences, corresponding to 25,920 steps (mean ± SD). In the last column, the values of the synthetic index ϵ.

Speed [km/h]	δ [-]	α [-]	γ [-]	ρ [-]	μ [-]	ϵ [-]
12.0	0.10 ± 0.11	0.02 ± 0.06	0.42 ± 0.13	0.44 ± 0.12	0.55 ± 0.19	0.48 ± 0.07
13.0	0.34 ± 0.25	0.22 ± 0.19	0.29 ± 0.13	0.31 ± 0.15	0.49 ± 0.16	0.45 ± 0.13
14.0	0.63 ± 0.26	0.62 ± 0.31	0.14 ± 0.11	0.20 ± 0.13	0.42 ± 0.14	0.38 ± 0.14
14.5	0.75 ± 0.26	0.79 ± 0.33	0.08 ± 0.10	0.13 ± 0.09	0.40 ± 0.15	0.36 ± 0.12

**Figure A1:**
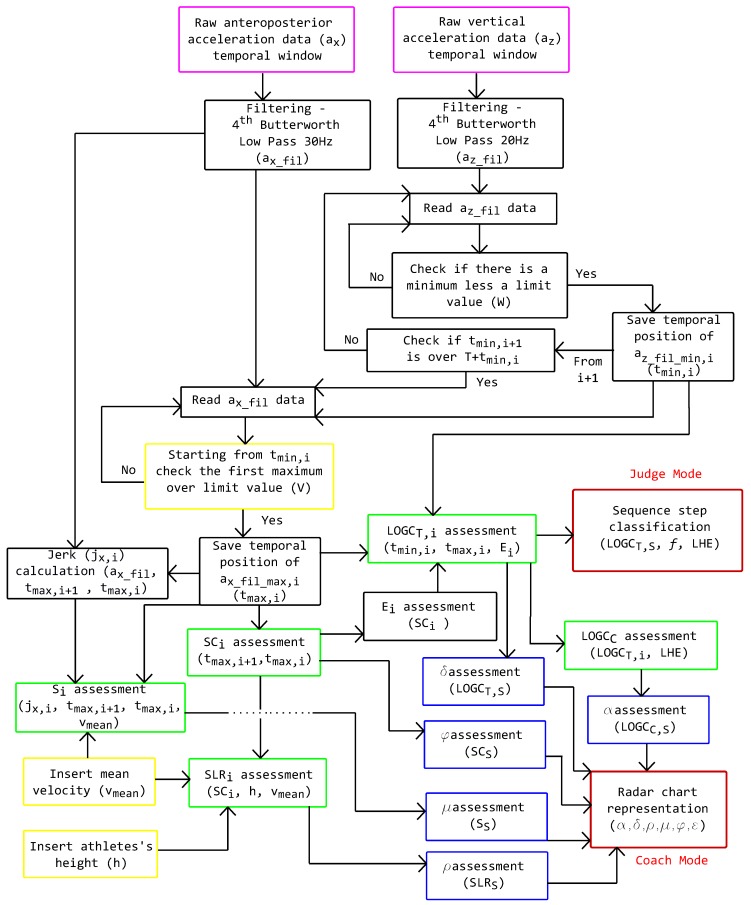
Flow chart of the data processing and analysis.

**Figure A2:**
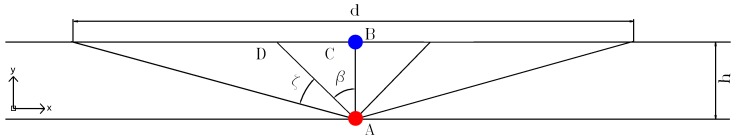
Geometric representation of the judge’s field of view. (A) Judge location; (B) Athlete location; (C) Area of observation for a field of view at β; (D) Additional Area for field of view allowed through acceptable eye rotation (ζ). The *x*-axis represents the athlete’s direction of progression; the *y*-axis is orthogonal to the athlete’s direction of progression.

## Abbreviations

The following abbreviations, symbols, and subscript are used in this manuscript:

AC	Acceptable Classification	α	biomechanical index related to $LOGC_{C,S}$
AUC	Area Under Curve	β	observation angle by the judges
ES	Effect Size	γ	biomechanical index related to SC
FN	Frame Number	δ	biomechanical index related to $LOGC_{T}$
FPR	False Positive Rate	Δ	Difference between Lee and P in statistics
FR	Frame Rate	ϵ	overall synthetic gesture index
HSE	Heel Strike Event	ζ	acceptable eye rotation
LAR	Least Angle Regression	η	fuzzy membership function of inertial sensor
Lee	Lee’s approach	θ	output value step classification functions
LHE	Limit of Human Eye	κ	specific weight of biomechanical index
LOGC	Loss Of Ground Contact	λ	fuzzy membership function of Benchmark
$LOGC_{B}$	Loss Of Ground Contact timing Benchmark	μ	biomechanical index related to S
$LOGC_{B,S}$	Loss Of Ground Contact timing Benchmark Sequence	ρ	biomechanical index related to SLR
$LOGC_{C,S}$	Loss Of Ground Contact Classification	σ	difference between η and λ
$LOGC_{T}$	Loss Of Ground Contact Timing	τ	percentage of acceptable classification
$LOGC_{T,S}$	Loss Of Ground Contact Timing Sequence	χ	function binary classification
MAPA	Maximum AnteroPosterior Acceleration	A	Area of the polygon area in radar chart
MD	Mean Difference in LOGC timing assessment	D	Doubt step
NPVA	Negative Peak Vertical Acceleration	E	Threshold for LOGC assessment
NS	Number of Steps	H	Hedges’ g coefficient value
OT	Optimal Threshold	I	Illegal step
PB	Personal Best	L	Legal step
PPV	Predict Positive Value	P	Proposed approach
ROC	Receiver Operating Characteristic	S	Smoothness
SC	Step Cadence	$S_{S}$	Smoothness Sequence
$SC_{S}$	Step Cadence Sequence	d	length of judge’s field of view
SD	Standard Deviation	f	sample frequency of inertial sensor
SL	Step Length	h	distance of the judge from race line
SLR	Step Length Ratio	j	jerk of the anteroposterior linear movement
$SLR_{S}$	Step Lenght Ratio Sequence	$t_{max}$	temporal instant of MAPA
TOE	Toe Off Event	$t_{min}$	temporal instant of NPVA
TPR	True Positive Rate	$v_{E}$	mean speed entry time world championship
WC	Wrong Classification	$v_{R}$	mean speed world record 20 km men

## Appendix A

Figure A1 reports a flowchart of the data processing and analysis method described in Section 2.2 and Section 2.3. The output for the two modes of operations (i.e., judge and coach modalities) are outlined by red boxes. The raw data collected by the measurement unit are placed in the violet box on the top. The performance and infringement parameters are inside the green boxes, while the related customized biomechanical indices are within the blue boxes. Finally, in the yellow boxes the additional data to be inserted by users in the operation mode “coach” are given. This picture provides a graphical summary of the methodology described in this paper.

## Appendix B

The World Athletic regulations state that judges in race walking must consider a sequence of steps instead of a single step, for identification of athletes’ infringements. In this appendix, we calculate the number of steps (NS) that we consider, starting from the estimation of the judge’s field of view. The length of the judge’s field of view *d* is estimated as

(A1) d=tan(β+ζ)·2h

where β is the angle to observe the athletes with respect to the race walking direction, ζ is the acceptable eye rotation, and *h* is the distance of the judges from the race line. For a geometrical interpretation of these parameters, see Figure A2. We use the following values: β = 45° and *h* = 5.50 m, as recommended by World Athletics for the judging [1]; ζ = 30°, as suggested by Shimizu [26].

With respect to the speed, the step length (SL) of a single athlete’s step varies in a range. We evaluate the number of steps (NS) to be included to define a proper sequence of steps as

(A2) $$NS=\mathrm{round}\left(\frac{\tan(\beta +\zeta )\cdot 2h}{\max(\mathrm{SL})}\right)$$

where we indicate with round(·) the approximation of (·) to the nearest integer. According to the maximum value of SL in elite race walkers (~1.40 m [15]), we fixed NS equal to NS = 30.

## Appendix C

In this appendix, we report the confusion matrices of LOGC step binary classification (Table A1 and Table A2), LOGC step sequence binary classification (Table A3), and three-level classification for step sequences (Table A4). These confusion matrices are used in Section 3.2 for deriving the results of LOGC classification using the method proposed in this paper.

**Table A1:** Confusion matrix for the step binary classification of Lee’s $LOGC_{T}$, for tests at 12.0 km/h, 13.0 km/h, 14.0 km/h, 14.5 km/h, and 15.5 km/h. *Legal_B_* and *Illegal_B_* refer, respectively, to “legal” and “illegal” steps as classified from camera data. *Legal_I,Lee_* and *Illegal_I,Lee_* refer, respectively, to “legal” and “illegal” steps as classified from inertial data, following the Lee’s approach.

	*Legal_I,Lee_*	*Illegal_I,Lee_*	*Total*
12.0 km/h			
*Legal_B_*	488	41	529
*Illegal_B_*	7	4	11
*Total*	495	45	540
13.0 km/h			
*Legal_B_*	249	80	329
*Illegal_B_*	16	15	31
*Total*	265	95	360
14.0 km/h			
*Legal_B_*	167	84	251
*Illegal_B_*	24	85	109
*Total*	191	169	360
14.5 km/h			
*Legal_B_*	25	48	73
*Illegal_B_*	44	63	107
*Total*	69	111	180
15.5 km/h			
*Legal_B_*	0	11	11
*Illegal_B_*	0	169	169
*Total*	0	180	180

**Table A2:** Confusion matrix for the step binary classification of the proposed $LOGC_{T}$, for tests at 12.0 km/h, 13.0 km/h, 14.0 km/h, 14.5 km/h, and 15.5 km/h. *Legal_B_* and *Illegal_B_* refer, respectively, to “legal” and “illegal” steps as classified from camera data. *Legal_I,P_* and *Illegal_I,P_* refer, respectively, to “legal” and “illegal” steps as classified from inertial data, following the proposed approach.

	*Legal_I,P_*	*Illegal_I,P_*	*Total*
12.0 km/h			
*Legal_B_*	468	61	529
*Illegal_B_*	6	5	11
*Total*	474	66	540
13.0 km/h			
*Legal_B_*	181	148	329
*Illegal_B_*	4	27	31
*Total*	185	175	360
14.0 km/h			
*Legal_B_*	90	161	251
*Illegal_B_*	14	95	109
*Total*	104	256	360
14.5 km/h			
*Legal_B_*	34	39	73
*Illegal_B_*	57	50	107
*Total*	91	89	180
15.5 km/h			
*Legal_B_*	0	11	11
*Illegal_B_*	1	168	169
*Total*	1	179	180

**Table A3:** Confusion matrices for the step sequence binary classification, based on Lee’s (left, subscript *Lee*) and proposed (right, subscript *P*) approaches. *Legal_B_* and *Illegal_B_* refer, respectively, to “legal” and “illegal” steps as classified from camera data. *Legal_I,Lee_* and *Illegal_I,Lee_* refer respectively to “legal” and “illegal” steps as classified from inertial data, following the Lee’s approach. *Legal_I,P_* and *Illegal_I,P_* refer, respectively, to “legal” and “illegal” steps as classified from inertial data, following the proposed approach.

	*Legal_I,Lee_*	*Illegal_I,Lee_*	*Total_I,Lee_*	*Legal_I,Lee_*	*Illegal_I,Lee_*	*Total_I,Lee_*
*Legal_B_*	27	16	43	37	6	43
*Illegal_B_*	0	11	11	1	10	11
*Total*	27	27	54	38	16	54

**Table A4:** Three multi-class confusion matrices for the three-levels classification on step sequences. On the left, the values for the Lee’s approach; on the right, the values for the proposed approaches. *Legal_B_*, *Doubt_B_*, and *Illegal_B_* refer, respectively, to “legal”, “doubt”, and “illegal” steps as classified from camera data. *Legal_I,Lee_*, *Doubt_I,Lee_* and *Illegal_I,Lee_* refer respectively to “legal”, “doubt”, and “illegal” steps as classified from inertial data, following the Lee’s approach. *Legal_I,P_*, *Doubt_I,P_* and *Illegal_I,P_* refer respectively to “legal”, “doubt”, and “illegal” steps as classified from inertial data, following the proposed approach.

	*Legal_I,Lee_*	*Doubt_I,Lee_*	*Illegal_I,Lee_*	*Total_I,Lee_*	*Legal_I,P_*	*Doubt_I,P_*	*Illegal_I,P_*	*Total_I,P_*
*Legal_B_*	8	34	0	42	23	19	0	42
*Doubt_B_*	0	5	1	6	0	5	1	6
*Illegal_B_*	0	0	6	6	0	0	6	6
*Total*	8	39	7	54	23	24	7	54
